# Investigation of potential safety hazards during medical waste disposal in SARS-CoV-2 testing laboratory

**DOI:** 10.1007/s11356-021-13247-4

**Published:** 2021-03-06

**Authors:** Jun Lv, Jin Yang, Juan Xue, Ping Zhu, Lanfang Liu, Shan Li

**Affiliations:** 1 Taihe Hospital, Hubei University of Medicine, Shiyan, 44200 Hubei China; 2 Shiyan Center for Disease Control and Prevention, Shiyan, 442000 Hubei China; 3 College of Life Science and Technology, Huazhong Agriculture University, Wuhan, 430070 Hubei China; 4 College of Biomedicine and Health, Huazhong Agricultural University, Wuhan, 430070 Hubei China

**Keywords:** SARS-CoV-2, Biosafety, Medical waste, Re-contamination, Safety hazards, Prevention

## Abstract

This study aims to investigate the potential safety hazards and provide reference for improving the medical waste disposal procedure in SARS-CoV-2 testing laboratory. Our SARS-CoV-2 testing group detected the RNA residue on the surface of medical waste with Droplet Digital PCR, and held a meeting to discuss the risks in the laboratory medical waste disposal process. After effective autoclaving, SARS-CoV-2 contaminated on the surface of medical waste bags was killed, but the average concentration of viral RNA residues was still 0.85 copies/cm^2^. It would not pose a health risk, but might contaminate the laboratory and affect the test results. When the sterilized medical waste bags were transferred directly by the operators without hand disinfection, re-contamination would happen, which might cause the virus to leak out of the laboratory. Furthermore, we found that sterilization effect monitoring and cooperation among operators were also very important. In summary, we investigated and analyzed the potential safety hazards during the medical waste disposal process in SARS-CoV-2 testing laboratory, and provided reasonable suggestions to ensure the safety of medical waste disposal.

## Introduction

The COVID-19 epidemic has already spread around the world (Ali et al. 2020; Liu et al. 2020). Severe Acute Respiratory Syndrome Coronavirus 2 (SARS-CoV-2) as the causative agent of COVID-19 is highly infectious and lethal (Harrison et al. 2020, Koff and Williams 2020, Phua et al. 2020). The continuous emergence of variants such as D614G, Spike Y839, and 20A.EU1 make it spread faster (Baric 2020, Borges et al. 2020, Hodcroft et al. 2020, Kirby 2021).

Laboratory testing of SARS-CoV-2 is allowed to be performed in the Biosafety Level 2(BSL-2) laboratory, which is generating a large amount of medical waste every day, including discarded samples, reagents, consumables, and personal protective equipment (Saadat et al. 2020). Proper disposal of medical waste is a key to ensure laboratory safety and test quality. According to the laboratory biosafety guidance related to COVID-19 (WHO 2020), waste generated in the BSL-2 laboratory must be autoclaved, then transferred out through the transfer window, and handed over to a professional medical waste recycling department for incineration and destruction. However, the guide does not mention the process of transferring decontaminated waste out of the laboratory (WHO 2020). There may be some potential safety hazards in the waste disposal of the SARS-Cov-2 test laboratory, but few studies have evaluated about them. In this study, we tried to detect the denaturation effect of autoclaving on SARS-Cov-2 RNA by Droplet Digital PCR (ddPCR), investigate the risk factors in the process of medical waste transfer, and provide reasonable suggestions for prevention.

## Methods

### Sample collection

**Fig. 1:**
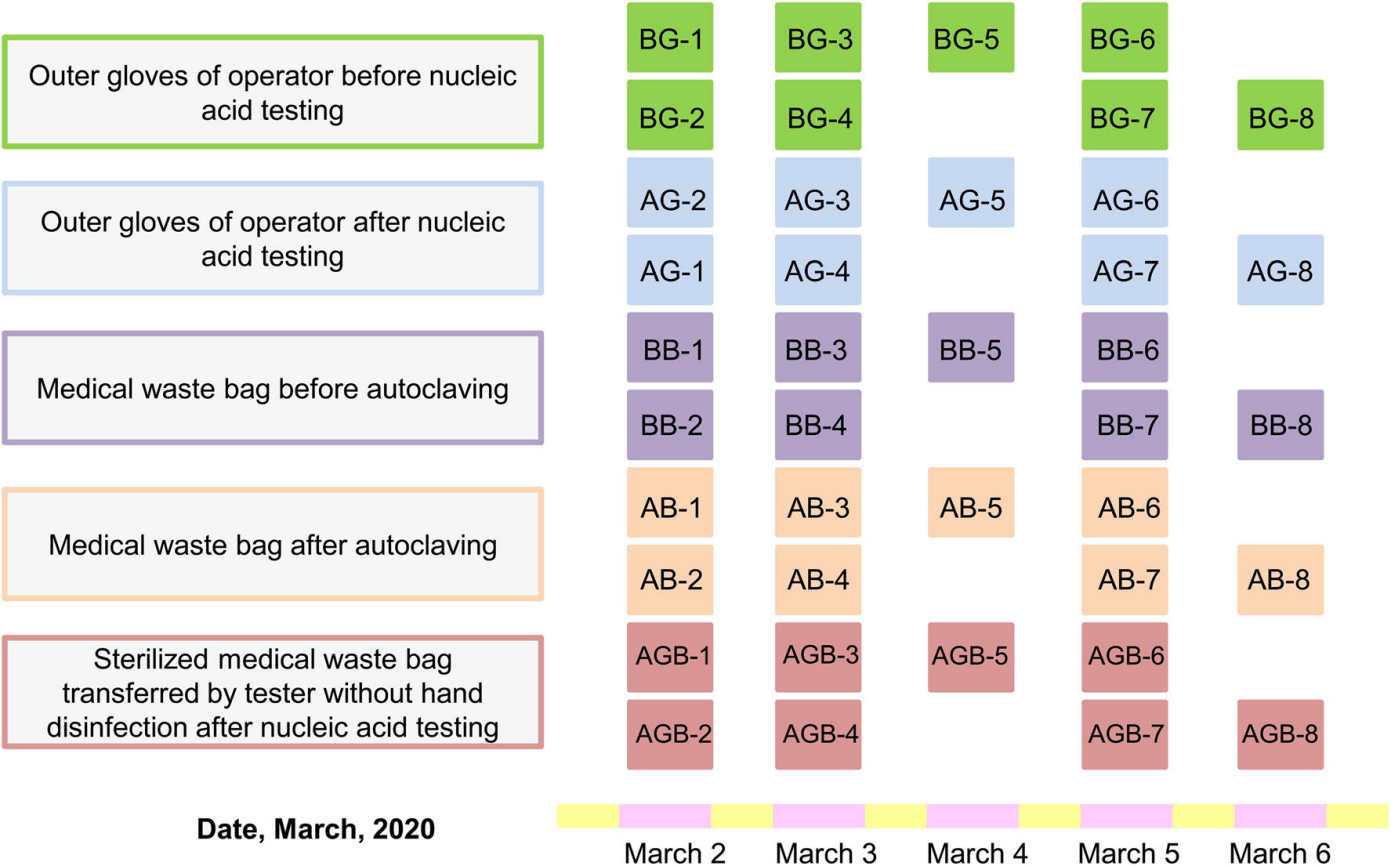
Sampling time and sample types

The Taihe Hospital in Shiyan City, Hubei Province, China, was one of the first hospitals to carry out nucleic acid detection of SARS-CoV-2. The sampling of medical waste was conducted from March 2 to March 6, as shown in Fig. 1. On March 2, 3, and 5, two batches of nucleic acid test were carried out, and each type of samples was collected twice. On March 4 and 6, only one batch of nucleic acid test was conducted, and each type of sample was collected once. The sampling area of each medical waste bag was 100 cm^2^ including four areas 5 cm × 5 cm. For gloves, the entire surface of fingers was sampled, and the sampling area was estimated. The samples indicated by BG, AG, BB, AB, and AGB were collected from outer gloves of operator before nucleic acid testing, outer gloves of operator after nucleic acid testing, medical waste bags before autoclaving, medical waste bags after autoclaving, and sterilized medical waste bags transferred by testers without hand disinfection after nucleic acid testing, respectively (Fig. 1).

### ddPCR detection

After sampling, all the samples were detected immediately in BSL-2 laboratory. Following the manufacturer’s instruction, viral RNA was extracted using a Viral Nucleic Acid Isolation Kit (Bioperfectus, Cat: SDK60102). Then, the ddPCR was used to amplify the specific target genes (*ORF1ab* and *N*) of SARS-CoV-2 on the Bio-rad QX200 system. The reaction mixtures and conditions were followed by a previous report (Lv et al. 2020). Finally, the output data was analyzed with the Quanta Soft TM analysis software, and the concentration calculation was calculated by Poisson distribution.

### Analysis of risk factors in the medical waste transfer

During the COVID-19 outbreak, multiple groups of operators took turn to carry out nucleic acid detection every day. Meanwhile, the medical waste generated from each batch need to be safely and properly disposed. Based on the actual operating experience, a nucleic acid testing team consisting of 11 people in this hospital held a meeting to discuss the risks in the process of sterilization, transfer, and handover of medical waste.

## Results

### Denaturation effect of autoclaving on SARS-CoV-2 RNA

The test results of SARS-CoV-2 RNA residues on the surface of medical waste bags before and after autoclaving were shown in Table 1. Before autoclaving, all the surface of medical waste bags were positive for SARS-CoV-2 RNA and the concentration ranged from 16.80 to 37.80 copies/cm^2^, with an average of 22.84 copies/cm^2^. After autoclaving at 103.4 mPa, 121.3 °C for 28 min, 5 out of 8 samples were positive, and the average concentration of SARS-CoV-2 RNA was 0.85 copies/cm^2^.

**Table 1:** List of sample information and detection results

Sampling stage	Sample type	Sample number	Sampling area (cm^2^)	Concentration (copies/cm^2^)
Before nucleic acid test	Outer gloves of operator	BG-1	73	0.00
		BG-2	73	0.00
		BG-3	73	0.00
		BG-4	73	0.00
		BG-5	73	0.00
		BG-6	73	0.00
		BG-7	73	0.00
		BG-8	73	0.00
After nucleic acid test	Outer gloves of operator	AG-1	73	51.78
		AG-2	73	14.96
		AG-3	73	15.25
		AG-4	73	20.14
		AG-5	73	11.79
		AG-6	73	9.06
		AG-7	73	6.04
		AG-8	73	27.33
Before autoclaving	Medical waste bag	BB-1	100	16.80
		BB-2	100	23.10
		BB-3	100	23.10
		BB-4	100	25.20
		BB-5	100	17.85
		BB-6	100	21.00
		BB-7	100	37.80
		BB-8	100	17.85
After autoclaving	Medical waste bag	AB-1	100	0.00
		AB-2	100	1.79
		AB-3	100	0.00
		AB-4	100	0.00
		AB-5	100	0.84
		AB-6	100	1.05
		AB-7	100	0.84
		AB-8	100	2.21
After nucleic acid test, the tester directly transferred the sterilized medical waste without hand disinfection.	Medical waste bag	AGB-1	100	5.78
		AGB-2	100	1.68
		AGB-3	100	3.57
		AGB-4	100	4.73
		AGB-5	100	1.79
		AGB-6	100	4.10
		AGB-7	100	0.84
		AGB-8	100	4.41

The autoclave procedure was recommended by the instrument manufacturers. The sterilization effect biological indicator (Attest^TM^ 1262) used in this autoclaving did not change the color after incubating at 56 °C for 48 h, which showed that the sterilization was successful and all microorganisms had been killed. However, the outer surface of some medical waste bags were still positive for SARS-CoV-2 RNA, which indicated that autoclaving was designed to kill the virus, but it could not completely degrade the viral nucleic acid.

### Re-contamination of sterilized medical waste

**Fig. 2:**
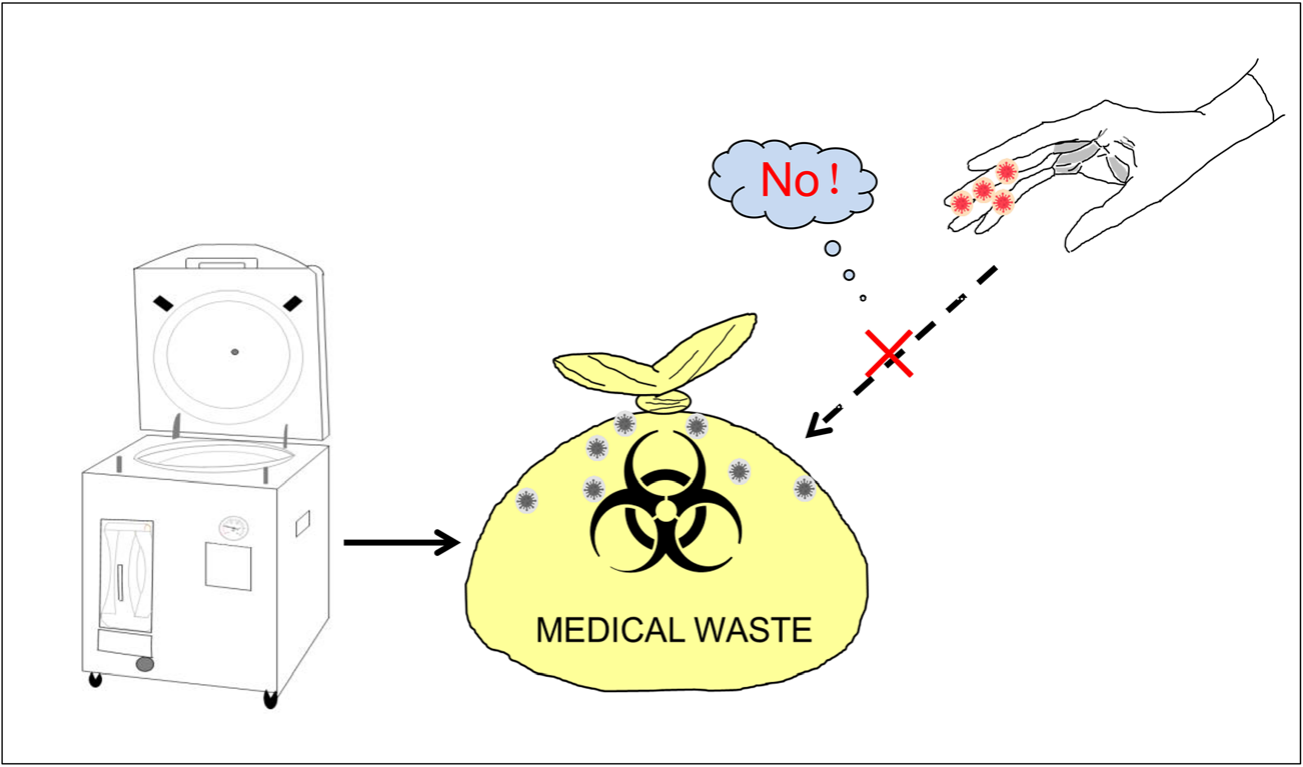
Medical waste being re-contaminated during transferring. The red and gray stars represented live and dead viruses, respectively

Before the nucleic acid test, 8 samples of tester’s outer gloves were negative for SARS-CoV-2 RNA. In contrast, after the nucleic acid test, all of them had been contaminated by the SARS-CoV-2 RNA, and the average concentration of SARS-CoV-2 RNA was 19.54 copies/cm^2^. When the medical waste bags were transferred after autoclaving by operators without hand disinfection, the surface of all the sterilized medical waste bags were positive for SARS-CoV-2 RNA again. The concentration ranged from 0.84 to 5.78 copies/cm^2^, and the average concentration of SARS-CoV-2 RNA increased from 0.85 to 3.36 copies/cm^2^. It indicated that the sterilized medical waste was re-contaminated by the operator’s gloves, as shown in Fig. 2. Considering that there might be live virus remaining on the surface of the tester’s gloves, it might cause the virus to leak out of the laboratory in the medical waste transfer process.

### Risk factors during medical waste handover process

After group discussion, we found that there were several risks in the process of medical waste disposal in SARS-CoV-2 testing laboratory.

**Fig. 3:**
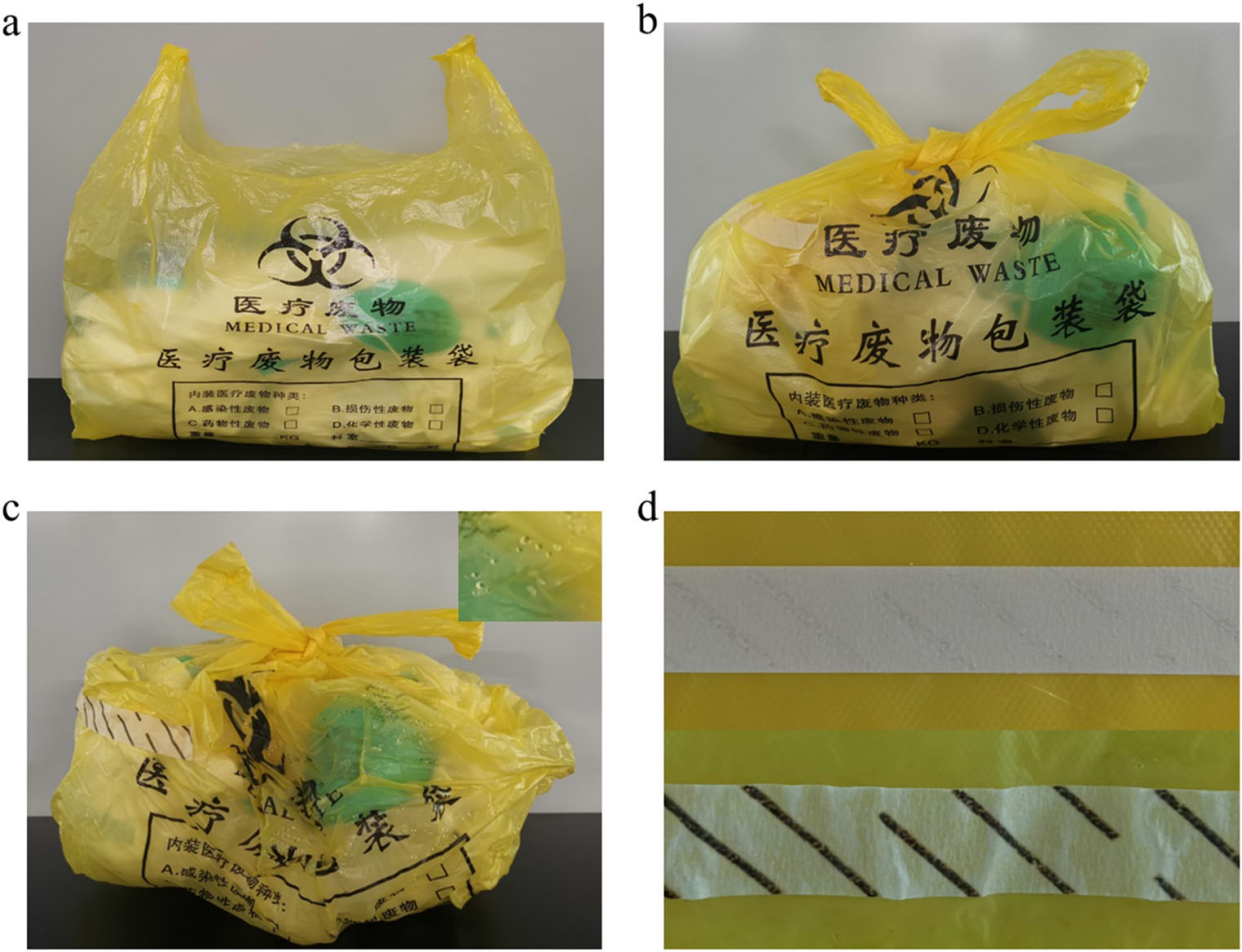
Observation points of medical waste after autoclaving. a The medical waste bag is not tied. b The shape of the medical waste bag is intact before autoclaving. c After autoclaving, the mouth of the medical waste bag must be tied tightly to prevent re-contamination, and obvious shrinkage and water droplets on the inner wall can be observed. d Comparison of sterilization effect indicator tape before and after autoclaving. The color of the sterilization effect indicator tape changes from light to dark brown, after autoclaving

1. In the BSL-2 laboratory, non-strict sterilization effect monitoring may increase the risk of virus leakage through medical waste transfer.
2. During the COVID-19 outbreak, multiple groups of testers have to perform nucleic acid test in batches every day to screen large cluster of patients. Poor communication and cooperation among operators in medical waste disposal may be also a risk.
3. In negative pressure laboratory, physical discomfort caused by wearing personal protective equipment and high-intensity labor may increase the error probability in the handover of medical waste. However, the post-processing personnel outside the laboratory usually do not care about the changes in the appearance of the medical waste after autoclaving (Fig. 3). So, mistakes in the transfer of medical waste cannot be discovered in time.

## Discussion

Approximately 10% of typical medical waste is infectious (Chartier et al. 2014). Incineration, chemical disinfection, and physical disinfection are commonly used for hospital waste disinfection (Ilyas et al. 2020; Wang et al. 2020). Improper medical waste management increases the potential for COVID-19 spread, especially in developing countries (Nzediegwu and Chang 2020). Safe and effective medical waste management is one of China’s experiences in successfully controlling the COVID-19 epidemic (Ma et al. 2020, Singh et al. 2020). All the medical waste generated in SARS-CoV-2 testing laboratory are considered infectious; this study exclusively focused on the risks of laboratory medical waste disposal.

Autoclaving is the most efficient way to sterilize the medical waste. In this article, the spore population of Attest^TM^ 1262 used for sterilization effect monitoring is 4.0 × 10^5^. After autoclaving, they could not be revived by culture, which indirectly indicates that all viruses contaminated on medical waste had been inactivated. The genome of a virus is DNA or RNA, which is more susceptible to be damaged under hydrated conditions than in dry conditions (Choi et al. 2014). Autoclaving may take 2 h to effectively eliminate nanogram quantities of contaminating nucleic acid (Gefrides et al. 2010). However, the maintenance time of autoclaving at 121 °C is generally about 30 min, which is not enough to completely degrade the nucleic acid of pathogenic microorganisms. Studies have shown that after autoclaving, shorter DNA or RNA fragments produced by incomplete degradation of viral nucleic acid may be recovered by molecular amplification techniques (Choi et al. 2014; Unnithan et al. 2014). Our results also showed that autoclaving could not completely degrade the viral nucleic acid. After autoclaving, the SARS-CoV-2 was killed and did not pose a health risk. However, the viral RNA residue might contaminate the laboratory environment and affect subsequent test results. The real-time quantitative PCR testing of SARS-CoV-2 targeted one or more genes, such as *ORF1ab* and *N* (Mathuria and Yadav 2020). If there was a target gene sequence in the nucleic acid residue, a false-positive result would be obtained. Therefore, after sterilization, medical waste should not be accumulated in the laboratory, and must be removed from the laboratory as soon as possible to prevent the residual nucleic acid from contaminating the laboratory environment and affecting the test results.

Disinfection or replacement outer gloves before transferring sterilized medical waste are just a small detail. In this study, if the operator did not comply, sterilized medical waste was re-contaminated. There might be live virus remaining on the surface of the tester’s gloves, which might cause the virus to leak out of the laboratory. Therefore, we recommend that in biosafety laboratory, operators should disinfect or replace outer gloves before transferring sterilized medical waste to prevent re-contamination. Laboratory managers can put a warning sign on the lid of the autoclave to remind the operators to avoid the re-contamination of medical waste as much as possible.

Sterilization effect monitoring is very important for ensuring laboratory biosafety. Especially in developing countries, there is a high proportion of sterilization failure (Panta et al. 2019a). In addition to qualified sterilization facilities and strict compliance with operating procedures, a proper sterilization effect monitoring method is essential. Medical waste can only be transferred out of the laboratory after passing the sterilization effect monitoring. There are biological indicators and chemical indicators can be used to monitor the sterilization effect of autoclaving. Biological indicator monitoring is the safest and most reliable method for measuring the effectiveness of autoclaving (Panta et al. 2019b), but it cannot show the sterilization effect at the end of autoclaving (Garibaldi et al. 2017). The conventional biological indicators Attest^TM^ 1262, Proof Plus, Assert, and Biosign usually need to be incubated at 56 °C for 48 h to get the monitoring result (Skaug and Berube 1983). The rapid Readout biological indicator Attest^TM^ 1292 is equivalent to conventional biological indicators and needs to be placed in a fluorimetric auto-reader for 3 h to detect fluorescence (Rutala et al. 1996). Chemical indicators mainly monitor the process of autoclaving through the color changes (Jabbari et al. 2012). Six classes of chemical indicators have been defined by the ANSI/AAMI/ISO 11140-1:2005 Standard (Puttaiah et al. 2014). Class 1 and class 2 chemical indicators only indicate whether the waste in the autoclave has gone through a sterilization cycle by color changes, but cannot assess the effectiveness of autoclaving. Class 3 and class 4 chemical indicators only measure one and two parametric variable (such as temperature, pressure, and maintenance time) in autoclaving process. Class 5 and class 6 chemical indicators are integrators and theoretically expected to be equivalent to biological indicators in terms of assessing effectiveness of autoclaving.

The best sterilization effect monitoring program is to use biological indicators for regular and periodic monitoring of autoclaves, and use chemical indicators for monitoring each sterilization cycle (Puttaiah et al. 2014). However, the quality and quantity of sterilization effect indicators, using regulations and enforcement, vary in different countries, which lead to different levels of sterilization effect monitoring failure. SARS-Cov-2 is too contagious; any negligence in the process of medical waste disposal in the testing laboratory can cause virus leakage or even personnel infection. Therefore, we recommend that the SARS-CoV-2 testing laboratory develop a strict sterilization effect monitoring program, use qualified biological indicators and chemical indicators, and implement them strongly. The autoclaves should be monitored with biological indicators at least once a week, or even daily when conditions permit. For each autoclaving cycle, a class 1 chemical indicator (autoclave indicator tape) and a class 5 chemical indicator can be used simultaneously. Medical waste will be moved from autoclaves to transfer window only when class 5 chemical indicator showed “ACCEPT.” Autoclave indicator tapes, pasted on the surface of medical waste bags, can help subsequent processing personnel outside the BSL-2 laboratory to determine that the medical waste has gone through autoclaving cycle.

During the COVID-19 outbreak, multiple groups of testers have to perform nucleic acid test in batches every day to screen large clusters of patients. The latter batch of testers often needs to help the former batch of testers transfer the autoclaved medical waste, or even complete the autoclaving. Personal protective equipment can protect the operators from infection (Cook 2020), but the air permeability is poor. Especially in the BSL-2 laboratory with negative pressure, operators often feel stuffy and breathless, hoping to complete the test and leave the laboratory as soon as possible, which will increase the risk of errors in the medical waste disposal process. To reduce the risk, we recommend that the medical waste transfer process in the BSL-2 laboratory operation guide needs to be improved, so that the operators can cooperate closely in medical waste treatment. In addition, the post-processing personnel outside the BSL-2 laboratory should carefully observe the appearance of medical waste before handing over and receiving. Once unsterilized medical waste is found, the transfer should be stopped immediately.

This article also has some limitations. During the COVID-19 outbreak in this city, all our energies were focused on carrying out nucleic acid testing to screen new confirmed cases from fever patients, suspicious cases, and close contacts. Since March 1, no new confirmed cases had been found, and we mainly conducted discharge testing of confirmed cases and environmental monitoring in public places. Then, we took the time to investigate the potential safety hazards during medical waste disposal in SARS-CoV-2 testing laboratory. The sampling time range and the sample size were small, and no statistical reasoning had been provided. Furthermore, in some developing countries with poor economic conditions, there is a lack of standard BSL-2 laboratories, qualified autoclave facilities, even personal protective equipment in primary hospitals. For them, the SARS-CoV-2 testing laboratory faces more risks in the medical waste treatment process, and further research is needed.

## Conclusions

Proper disposal of medical waste is a key to the laboratory safety. In this study, we found that there were some potential safety hazards during medical waste disposal in the SARS-CoV-2 testing laboratory. Autoclaving cannot completely degrade the viral genome, so sterilized medical waste should be transfer out of the laboratory as soon as possible to prevent nucleic acid contamination. Operators must disinfect or replace outer gloves before transferring sterilized medical waste to prevent re-contamination. The SARS-CoV-2 testing laboratory need to develop a strict sterilization effect monitoring program, use qualified biological indicators and chemical indicators, and implement them strongly. In the process of medical waste transfer and handover, close cooperation between operators and careful observation of follow-up processing personnel outside the laboratory can reduce the risk of virus leakage due to mistakes.

The above findings and recommendations can alert the health authorities to pay more attention to laboratory biosafety management, especially to improve the laboratory medical waste treatment process. Eliminating potential safety hazards is able to protect the internal and external environment of the laboratory from contamination, which will ensure the safety and health of laboratory operators and surrounding personnel.

## Data Availability

Not applicable
